# Variance and Covariance of Actual Relationships between Relatives at One Locus

**DOI:** 10.1371/journal.pone.0057003

**Published:** 2013-02-22

**Authors:** Luis Alberto Garcia-Cortes, Andres Legarra, Claude Chevalet, Miguel Angel Toro

**Affiliations:** 1 Departamento de Mejora Genética Animal, INIA, Madrid, Spain; 2 INRA, UR 631 SAGA, Castanet-Tolosan, France; 3 INRA, UMR 444 LGC, Castanet-Tolosan, France; 4 Departamento de Producción Animal, Universidad Politécnica de Madrid, Madrid, Spain; University of California, Riverside, United States of America

## Abstract

The relationship between pairs of individuals is an important topic in many areas of population and quantitative genetics. It is usually measured as the proportion of thegenome identical by descent shared by the pair and it can be inferred from pedigree information. But there is a variance in actual relationships as a consequence of Mendelian sampling, whose general formula has not been developed. The goal of this work is to develop this general formula for the one-locus situation,. We provide simple expressions for the variances and covariances of all actual relationships in an arbitrary complex pedigree. The proposed method relies on the use of the nine identity coefficients and the generalized relationship coefficients; formulas have been checked by computer simulation. Finally two examples for a short pedigree of dogs and a long pedigree of sheep are given.

## Introduction

The relationship between pairs of individuals is an important topic in many areas of population and quantitative genetics [1]. The degree of relationship is measured as the proportion of the genome identical by descent shared by the pair and can be inferred from pedigree information. But there is a variance in the realized, or actual, proportion of genome shared as a consequence of Mendelian sampling and linkage. For instance, two full-sibs can share zero, one or two alleles identical by descent (giving a variance of 1/2 in the number of alleles actually shared), whereas non inbred father and son share exactly one allele (variance of 0). Formulae have been published for the variance of actual relationship for a number of specific types of relatives (see [2] and references therein) but a general formula has not been developed.

Deviations of coancestry from the ideal situation of infinite unlinked loci cause linkage disequilibria across pairs of loci [3]. These disequilibria are used extensively nowadays for mapping regions controlling traits (e.g., by genome-wide association studies (GWAS)), genomic selection in crop plants and domestic animal populations, phasing of markers for imputation or quantitative trait locus detection, or control of stratification in GWAS through, for instance, principal component analysis. Therefore, a mathematical formulation of these deviations is critical for the understanding of modern methods of genetic analysis, even if they are based on molecular markers.

For instance, Powell et al. [4] suggested a “reconciliation” of identity by state (IBS, critical in GWAS studies) and identity by descent (IBD, used in pedigree analysis) through a notion of base population and the use of Wright’s F fixation indices. However, this assumes ideal populations. In the case of plant and animal breeding populations, pedigree is usually known.

For the simplest one locus situation the coancestry between two individuals is the probability that two alleles chosen at random, one from each individual, are identical by descent. The fraternity coefficient is defined as the probability that single locus genotypes (both genes) of two individuals are identical by descent. The purpose of this note is hence to develop the theory to predict the variances and covariances of realized coancestry and fraternity coefficients for pedigreed populations at a single locus. Here we develop a simple expression and algorithm to calculate the variance of these two coefficients and verify it through computer simulation.

## Materials and Methods

In this section we show how the moments of coancestries can be calculated from the identity coefficients developed by Harris [5] and Gillois [6], and the generalized kinship coefficients of Karigl [7].

### The Nine Condensed Identity States

In the genealogical analysis we consider ‘virtual’ genes that are all different in the founder population. In this setting, when ignoring the paternal or maternal origin, the relationship between two individuals for one locus can be described exhaustively with the nine condensed identity coefficients. The calculation of these coefficients from pedigrees is fairly well known [7].

**Figure 1:**
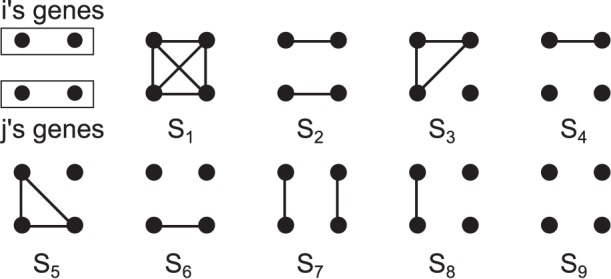
The nine condensed identity states. Upper dots represent both copies of individual “i” and lower dots represent both copies of individual “j”. Dots linked with lines are identical by descent and dots not linked are explicitly non identical.

Figure 1 shows the nine condensed identity states as they have been presented in the literature, starting from S_1_ (the four copies are identical by descent), to S_9_ (the four copies are non-identical). The probability of each state S_k_ is usually denoted as 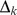.

The nine condensed identity coefficients express the identity given the segregation at the previous generation, for example, two full brothers whose parents were founders can be at the state S_7_ if they both received the same copy from the sire and the dam, they can be at state S_8_ if they received the same copy from the sire or the dam but not both. Finally they can be at state S_9_ if they received different copies from both the sire and the dam. The probabilities of these three states are easy to obtain based on Mendelian segregation rules: 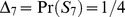,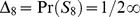 and 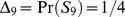. Identities of a founder parent-offspring case are even easier, because only 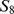 has a nonzero probability: 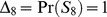.

In general, the states 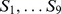 and the probabilities 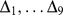 define a categorical distribution for each pair of individuals. Each independent locus observed in these two individuals is an independent realized value of this categorical distribution.

Using the Iverson brackets notation, the moments of the conventional categorical distribution for the identity states 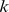,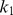 and 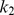 are:
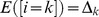


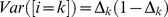
(1)

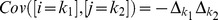
(2)where “*i = k*” is a probabilistic event meaning that the realized value for a given locus in a given pair of individuals is the state “*k*”, the Iverson variable 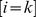 equals 1 if the event is true and 0 if the event is false.

### The Variance of the Coancestry Coefficient Accounting for Segregation

The expected coancestry coefficient between two individuals can be calculated from the condensed identity coefficients using the formula [5]

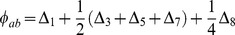
or
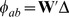
(3)Where 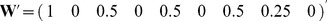 and 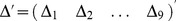.

When considering variance due to segregation, each constant 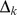 has to be replaced by the corresponding Iverson bracket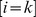. The variance of the relationship between two individuals due to the segregation can be easily obtained from formula 3 as:
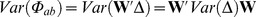



or,
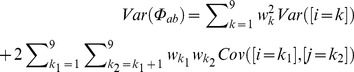
(4)(note that 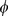 stands for expected coancestry and 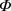 for realized or actual coancestry, i.e., given the Mendelian segregation). Replacing formulas (1) and (2) in (4) gives:
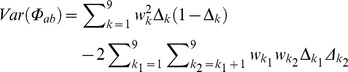
(5)which has up to 25 nonzero terms. Reordering the terms of formula 5,



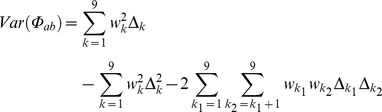


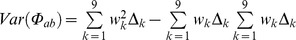
which can be easily expressed as a function of generalized kinship coefficients. Following Karigl’s [7] notation,
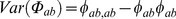
(6)where the term 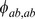 is a generalized coefficient of kinship for two pairs of individuals.

Covariances between coancestry relationship coefficients can be also derived from the generalized kinship coefficients. The proof is included in the Supplementary Material and the result is the obvious generalization of formula 6.
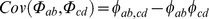



The number of covariances of the coancestry coefficients is very large. If there are 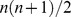 different coancestry coefficients, there are 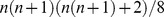 different covariances.

### The Variance of the Fraternity Coefficient Accounting for Segregation

The fraternity coefficient can be obtained from the condensed states of identity :
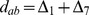



In this case, formula 5 can also be used but the correct weights must be set, that is, 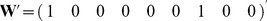. In this case 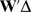 only has up to four nonzero elements.
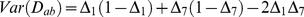
(note that *D* stands for realized fraternity and *d* for expected fraternity).

Formula 5, in fact, also holds for any linear aggregate of 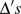. For instance, 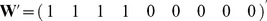 or 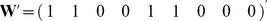
correspond respectively to inbreeding coefficients of individual *i* and *j*. A formula for the covariance of two fraternity coefficients is given in Appendix S1.

## Results

In this section, the coancestry of a pair of English Setter dogs is presented in order to illustrate the calculations and to compare the results with MonteCarlo simulations. Afterwards, the coancestries of 11 Latxa sheep were also analyzed in order to test the computational feasibility of the algorithms.

### Example. The Genetic Relationship between the Setters Dash 2nd and Moll 3rd

**Figure 2:**
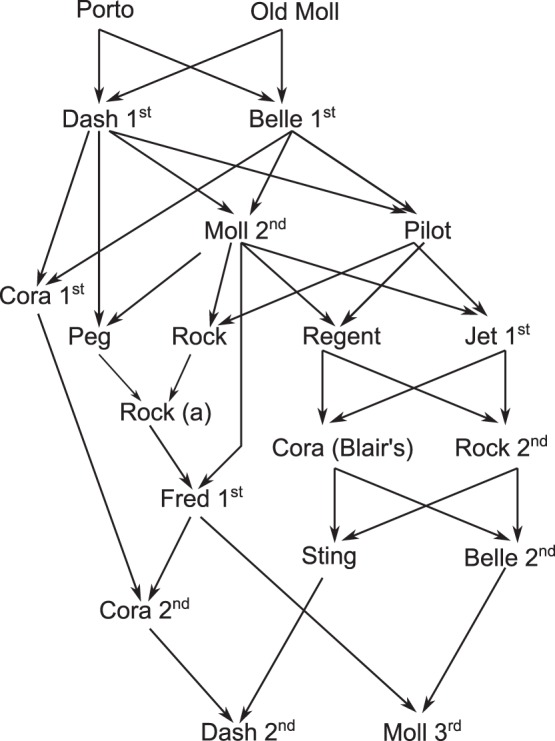
The pedigree of Dash 2^nd^ and Moll 3^rd^ .Two English setters breed by Edward Laverackin the middle of S. XIX.

During the XIX century, animal breeders sometimes planned in-and-in pedigrees to keep the blood “pure”. Edward Laverack [8] implemented the matings presented in Figure 2 to achieve “the perfection of form adapted to speed nose and endurance” required for hunting dogs. The intricacy of this ancient pedigree satisfies our testing purposes.

Sting and Belle 2^nd^, close relatives after 5 generations of full brother matings, were mated with Cora 2^nd^ and Fred 1^st^, collateral relatives. The resulting progeny, Dash 2^nd^ and Moll 3^rd^, are simultaneously half brothers and aunt-nephew and their close relatives are highly inbred. For that reason, the relationship between these two dogs has nine non-null condensed identity coefficients, that is
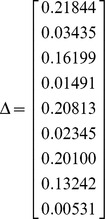



In Table 1 we present the coancestry coefficient and its variance calculated after formulae (3) and (4), as well as MonteCarlo estimates of coancestries and their the variances, obtained by gene dropping [9]. Both the theoretical values and the MonteCarlo estimates agree perfectly well.

**Table 1:** Exact relationships of Dash 2^nd^ and Moll 3^rd^ and their corresponding Montecarlo gene dropping estimates obtained with 100000 samples.

	W	Expectation	Variance	Montecarlomean	Montecarlo variance
Coancestry	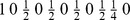	0.5371	0.0810	0.5367	0.0811
Fraternity	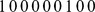	0.4194	0.2435	0.4169	0.2431
Inbreeding Dash 2^nd^	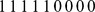	0.4297	0.2451	0.4309	0.2452
Inbreeding Moll 3^rd^	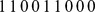	0.4844	0.2498	0.4845	0.2497

It is well known that the inbreeding of an individual is equal to the coancestry between his parents. Nevertheless, it is interesting to note that the variance of the actual inbreeding coefficient of an individual is not equal to the variance of the actual coancestry between his parents. For instance, the expectation of the inbreeding of Dash 2^nd^ is 0.4297 (Table 1) calculated from the condensed identity coefficients between Dash 2^nd^ and Moll 3^rd^. Although not included in Table 1, the coancestry between his parents, Cora 2^nd^ and Sting, calculated from their shared condensed identity coefficients, is obviously 0.4297. However, the variance of the actual inbreeding of Dash 2^nd^ is 0.2451, yet the variance of the coancestry between its parents is 0.0922. In general, the variance of the inbreeding will be equal or greater than the corresponding coancestry because it accumulates an extra step of Mendelian segregation.

It can be shown algebraically that the variance of the realized inbreeding coefficient F is *f*(1–*f*) (where *f* is the expected inbreeding coefficient) as it should be. In effect, by definition, the realized inbreeding is 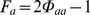. Applying formula 6 and the result presented in [7], i.e. 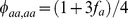, it turns out that 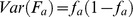.Results presented in lines 3 and 4 of Table 1 agree with that formula.

There is also a good agreement between the theoretical covariance between two coancestries and the values estimated by MonteCarlo simulation. For example, the covariance between the coancestry of the pair Cora 2^nd^ and Siting and the coancestry of the pair Dash 2^nd^ and Moll 3^rd^ has a theoretical value of 0.04188. The covariance between the coancestry of the pair Cora 2^nd^ and Sting and the coancestry of the pair Fred 1^st^ and Belle 2^nd^ is 0.04466. The corresponding MonteCarlo estimates for both covariances are 0.04179 and 0.04436.

### Example. Long Pedigree in Latxa Breed

A complex pedigree of 6175 animals of the Latxa sheep was analyzed. We computed the coancestries and variances and covariances of coancestries of the last eleven individuals (in renumbering order). This was an expensive task, for the recursions in Karigl’s method [7] required the computation of >340,000,000 coefficients. The results for four individuals are in Tables 2 and 3; the pedigrees of those four individuals are known for 9–11 generations (often incompletely: the number of equivalent complete generations (e.g. [10]) is respectively 2.85, 4.06, 4.34, 3.28). Individuals 1 and 8 are father and son; individuals 1 and 2 are slightly related. Neither 1 nor 9 are inbred. It can be seen that low relationships (e.g. animals 1 and 2) have proportionally higher variances, as shown by [2], whereas null relationships have a null variance. Interestingly, there are negative covariances among relationships. Covariances between realized coancestries are most often very small, except the covariances between very close ones, e.g. 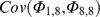, which is natural.

**Table 2:** Coancestries of four individuals in the Latxa sheep.

	1	2	8	9
1	0.5	0.0025	0.2543	0
2		0.5052	0.0037	0
8			0.5043	0
9				0.5

**Table 3:** Variances and covariances (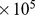 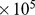) of all coancestries of four individuals in the Latxa sheep.

	(1,1)	(1,2)	(1,8)	(1,9)	(2,2)	(2,8)	(2,9)	(8,8)	(8,9)	(9,9)
(1,1)	0	0	0	0	0	0	0	0	0	0
(1,2)		66	−0.28	0	5.7	33	0	−0.29	0	0
(1,8)			100	0	0	−0.37	0	100	0	0
(1,9)				0	0	0	0	0	0	0
(2,2)					250	4.2	0	0	0	0
(2,8)						9.4	0	0.029	0	0
(2,9)							0	0	0	0
(8,8)								210	0	0
(8,9)									0	0
(9,9)										0

## Discussion

In this paper we have shown that variances and covariances of coancestries and inbreeding coefficients can be calculated analytically using the classical condensed identity coefficients. These tasks require using Karigl’s [7] double-pair coancestries. For small pedigrees or pedigrees with many generations, it is better to use tabular algorithms to obtain all double-pair coancestries, but in genealogies with a large number of individuals and a small number of generations, recursive function strategies were implemented to calculate only the coancestries required. An intermediate strategy (i.e., our method) was to store those coefficients that are being calculated for further use. Both approaches are computationally demanding because the number of double pair coancestries is *n* ^4^, where *n* is the number of individuals. However, in practice the number of computed coefficients may be much lower: 340×10^6^ relationships were computed in the Latxa example, against a possible total of 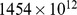.

The extension to several independent loci is straightforward. The categorical distribution in formulas (1) and (2) has to be replaced by the corresponding multinomial distribution, which basically consists in dividing variances and covariances by the number of loci. However, linkage affects variation in the actual identity coefficients between individuals with the same pedigree, and therefore increases its variance. The treatment of linkage is difficult and has been partially dealt with by several authors (see [2] and references therein). An interesting suggestion by Goddard [11], is to use the effective number of loci (M_e_) defined as the number of loci that provides the same variance of realized relationship as obtained in the more realistic situation. It can be calculated as 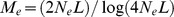 where 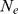 is the effective population size and *L* the gamete length in Morgan. Then, we simply divide the variances by this effective number of loci. In our example the variance of coancestry of Dash 2^nd^and Moll 3^rd^ (assuming a genome with 100 linked loci and a recombination fraction of 0.01 among adjacent loci) was 0.0204 (simulation results) and therefore, the effective number of loci is 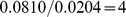 not far from the value obtained using Goddard’s formula (5.5 by using a 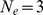).

Incomplete pedigrees will lead to estimate error. They will tend to bias coancestries and their coancestries downwards and increase their errors. For instance, two individuals with no recorded father will have null coancestries, and their common descendants (if any) will have smaller coancestries and covariances of the coancestries. Methods to deal with unknown paternities include either the use of uncertain paternities [12], or of pseudo-parents [13]. Rules to derive approximate covariances of coancestries may be obtained from those methods.

Instead of pedigree, markers are often used to derive relationships, for instance by computing measures of molecular coancestry and referring them to descent (e.g., [14]). These relationships are computed after observing the molecular state of the individual (not of their parents), i.e., the Mendelian sampling is somehow “observed”. So, molecular-based relationships do not suffer from sampling due to Mendelian sampling. They suffer, nevertheless, from lack of definition of allelic frequencies (i.e., which base population do we refer to?) and from sampling error due to the finite number of markers and linkage.

## Supporting Information

Appendix S1 **Covariances of two coancestries or fraternities.**
(PDF)

## References

[1] Lynch M, Walsh B (1998) Genetics and analysis of quantitative traits. Massachussets: Sinauer Associates. 980 pp.

[2] Hill WG, Weir BS (2011) Variation in actual relationship as a consequence of Mendelian sampling and linkage. Genet Res 93: 47–64. 10.1017/S0016672310000480 PMC3070763 21226974

[3] Hill WG, Weir BS (2007) Prediction of multi-locus inbreeding coefficients and relation to linkage disequilibrium in random mating populations. Theor Popul Biol 72: 179–185. 17575994 10.1016/j.tpb.2006.05.006 PMC2729754

[4] Powell JE, Visscher PM, Goddard ME (2010) Reconciling the analysis of IBD and IBS in complex trait studies. Nat Rev Gen 11: 800–805. 10.1038/nrg2865 20877324

[5] Harris DL (1964) Genotypic covariance between inbred relatives. Genetics 50: 1319–1348. 14239792 10.1093/genetics/50.6.1319 PMC1210738

[6] Gillois M (1964) La relation d’identité en génétique. Thesis, Faculté des Sciences de Paris.

[7] Karigl G (1981) A recursive algorithm for the calculation of identity coefficients. Ann Hum Genet 45: 299–305. 7305283 10.1111/j.1469-1809.1981.tb00341.x

[8] Laverack E (1872) The setter. Longmans, Green and co., London. 80 p.

[9] MacCluer JW, Vandeburg JL, Read B, Ryde OA (1986) Pedigree analysis by computer simulation. Zoo Biol. 5: 149–160.

[10] Boichard D (2002) PEDIG: a fortran package for pedigree analysis suited for large populations. Proceedings of the 7th World Congress on Genetics Applied to Livestock Production: 19–23 August 2002; Montpellier 2002, 28–13.

[11] Goddard M (2009) Genomic selection: prediction of accuracy and maximisation of long term response. Genetica 136: 245–257. 18704696 10.1007/s10709-008-9308-0

[12] Perez-Enciso M, Fernando RL (1992) Genetic evaluation with uncertain parentage: a comparison of methods Theor Appl Genet. 84: 173–179. 10.1007/BF00223997 24203044

[13] Colleau JJ, Sargolzaei M (2011) MIM: an indirect method to assess inbreeding and coancestry in large incomplete pedigrees of selected dairy cattle. J Anim Breed Genet 128: 163–173. 21554410 10.1111/j.1439-0388.2010.00899.x

[14] VanRaden PM (2008) Efficient methods to compute genomic predictions. J Dairy Sci 91: 4414–4423. 18946147 10.3168/jds.2007-0980

