# Supplementary material for: Variance and Covariance of Actual Relationships between Relatives at One Locus
Source: PLoS One. 2013 Feb 22;8(2):e57003. doi: 10.1371/journal.pone.0057003 (PMC3579841; doi:10.1371/journal.pone.0057003)
Supplement: Appendix S1 — Covariances of two coancestries or fraternities. (PDF) [file pone.0057003.s001.pdf]

Appendix . Covariances of two coancestries or fraternities

Consider the eight genes (1-8) in four individuals (A-D) as depicted in Supplementary Figure 1.

Supplementary Figure 1. Genes in four individuals.

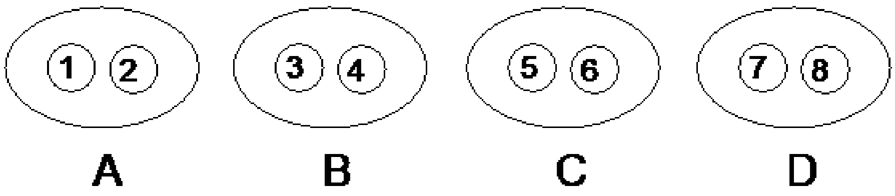

Realised coancestry between A and B is a function of the realised identity states:

$\Phi_{AB} = \frac{1}{4}([1 \equiv 3] + [1 \equiv 4] + [2 \equiv 3] + [2 \equiv 4])$, where "$\equiv$" within the Iverson brackets means identity by descent.

$E(\Phi_{AB}) = \Pr(\Phi_{AB} = 1) = \phi_{AB}$, the regular coancestry (by definition); we will use uppercase letters for realised identities or functions of them and lowercase letters for their expectations. This distinction is important because the identity states are either 0 or 1, their products are 0 or 1, and any expectation of these variables is a probability [S1]; making the algebra much simpler. Those properties disappear when working with probabilities of identity states.

The covariance is a function of cross-products of identity states:

$$
\begin{aligned}
\Phi_{AB}\Phi_{CD} &= \frac{1}{4}\left([1\equiv 3]+[1\equiv 4]+[2\equiv 3]+[2\equiv 4]\right)\frac{1}{4}\left([5\equiv 7]+[5\equiv 8]+[6\equiv 7]+[6\equiv 8]\right) \\
&= \frac{1}{16}\left([1\equiv 3][5\equiv 7]+[1\equiv 3][5\equiv 8]+\ldots[2\equiv 4][6\equiv 8]\right)
\end{aligned}
$$

This cross-product has 16 terms, but for our purposes it is enough to consider one example because the rest are probabilistically equivalent. Consider the product $[1\equiv 3][5\equiv 7]$. This equals 1 when "1" and "3" are identical by descent and *at the same time* "5" and "7" are identical by descent. That event happens with a certain probability, which is precisely $\Pr\left((1\equiv 3)\wedge(5\equiv 7)\right)=E\left([1\equiv 3][5\equiv 7]\right)$. This probability is 1/16 times Karigl's $\phi_{AB,CD}$ [7], which is the probability that two genes drawn from A and B are identical by descent and *at the same time* two genes drawn from C and D are identical by descent. The other 15 terms correspond to considering the other draws $[1\equiv 3][5\equiv 8]$, etc., which have the same probability. Therefore $E\left(\Phi_{AB}\Phi_{CD}\right)=\phi_{AB,CD}$ and thus

$$
\begin{aligned}
Cov\left(\Phi_{AB},\Phi_{CD}\right) &= E\left(\Phi_{AB}\Phi_{CD}\right)-E\left(\Phi_{AB}\right)E\left(\Phi_{CD}\right) \\
&= \phi_{AB,CD}-\phi_{AB}\phi_{CD}
\end{aligned}.
$$

This expression is, of course, valid for the particular case of $Cov\left(\Phi_{AB},\Phi_{AB}\right)=Var\left(\Phi_{AB}\right)$, as detailed in the main text. Because we deal with additive relationships, there is no need to include four-gene identities quantities, e.g., $\left([1\equiv 3\equiv 5\equiv 7]\right)$ which would require higher-order relationships.

Now consider realised dominant relationships *D*. They equal 1 (0) if the *genotypes* of A and B are (are not) identical by descent. This can be written as:

$D_{AB} = \frac{1}{2}\left([1 \equiv 3][2 \equiv 4] + [1 \equiv 4][2 \equiv 3]\right)$ and $E\left(D_{AB}\right) = \Pr\left(D_{AB} = 1\right) = d_{AB}$, the dominant relationship (or fraternity).

The cross-product $D_{AB}D_{CD}$ can be written as:

$$D_{AB}D_{CD} = \frac{1}{2}\left([1 \equiv 3][2 \equiv 4] + [1 \equiv 4][2 \equiv 3]\right)\frac{1}{2}\left([5 \equiv 7][6 \equiv 8] + [5 \equiv 8][6 \equiv 7]\right) =$$
$$\frac{1}{4}\left([1 \equiv 3][2 \equiv 4][5 \equiv 7][6 \equiv 8] + [1 \equiv 3][2 \equiv 4][5 \equiv 8][6 \equiv 7] + \right.$$
$$\left.[1 \equiv 4][2 \equiv 3][5 \equiv 7][6 \equiv 8] + [1 \equiv 4][2 \equiv 3][5 \equiv 8][6 \equiv 7]\right)$$

Again, consider one element, e.g. $\left([1 \equiv 3][2 \equiv 4][5 \equiv 7][6 \equiv 8]\right)$. This equals one when all four pairs of genes are identical by descent at the same time, but not necessarily identical among themselves. The probability of this event can be computed from the probabilities that the respective *gametes* forming A, B, C and D are identical by descent, and summing probabilities across all possible combinations. For instance, $D_{AB}D_{CD} = 1$ if the fathers of A and B transmitted the same gamete to A and B and *at the same time* the mothers transmitted (another) identical gamete to A and B and the same events happened for C and D; or, if the maternal and paternal alleles of A and B are identical and so are the paternal of A with the maternal of B; and so on. Therefore:

$$E\left(D_{AB}D_{CD}\right) = \Pr\left(D_{AB}D_{CD}\right) = \frac{1}{4}\left(\phi_{FA,FB;MA,MB;FC,FD;MC;MD} + \phi_{FA,MB;MA,FB;FC,FD;MC,MD}\right.$$
$$\left. + \phi_{FA,FB;MA,MB;FC,MD;MC,FD} + \phi_{FA,MB;MA,FB;FC,MD;MC,FD}\right)$$

.

Where *FA* (*MA*) is the father (mother) of A and $\phi_{A,B;C,D;E,F;G,H}$ is a four-individual generalization of $\phi_{AB,CD}$. These four-individual relationships are, in principle, computable [S2-S3]; but we did not attempt to do so.

Additional references

S1. Chevalet C (1971) Calcul a priori, intra-et inter-populations, des variances et covariances génotypiques entre apparentés quelconques. Gen Sel Evol 3: 463-477.

S2. Lange K (2002) Mathematical and statistical methods for genetic analysis. Springer Verlag, New York. 384 p.

S3. Abney M (2009) A graphical algorithm for fast computation of identity coefficients and generalized kinship coefficients. Bioinformatics 25: 1561-1563.
